# Study protocol for an adapted personal project analysis to measure vertical inter-goal relations on physical activity and diet

**DOI:** 10.1186/s40359-022-00931-4

**Published:** 2022-09-24

**Authors:** Sara Kassas, Catherine Culot, Geert Crombez, Annique Smeding, Christophe Leys, Debbe Thompson, Ann DeSmet

**Affiliations:** 1 Faculty of Psychology, Educational Sciences and Speech Therapy, Research Center for the Promotion of Health, Prosocial Behaviour and Well-Being (PACE), Université Libre de Bruxelles, Brussels, Belgium; 2 Faculty of Psychology and Educational Sciences, Department of Experimental Clinical and Health Psychology, University of Ghent, Ghent, Belgium; 3 Inter-university Laboratory of Psychology–Personality, Cognition, Social Change, Université Savoie Mont Blanc, Annecy, France; 4 U.S. Department of Agriculture/Agricultural Research Service Children’s Nutrition Research Center, Baylor College of Medicine, Houston, TX USA; 5 Department of Communication Studies, University of Antwerp, Antwerp, Belgium

**Keywords:** Personal project analysis, Personal values, Goal conflict and facilitation, Physical activity, Diet

## Abstract

**Background:**

The promotion of multiple healthy lifestyles has been implemented as part of public health efforts to prevent and reduce the burden of non-communicable diseases. However, these interventions have shown a heterogeneity in their effectiveness. The pursuit of multiple daily goals may influence overall progress in achieving health goals. Horizontal inter-goal relations can be conflicting (due to time constraints) or facilitating (due to goal compatibility) and impact progress towards goal achievement. Personal values also play an important role in health promotion. Personal values direct attention towards accomplishing a higher-level goal through goal setting. Identifying the conflicting or facilitating relationships between health goals and personal values would provide insights in how individuals value health and the personal values that may support the adoption of a healthy behavior. The health goals that this study will focus on are physical activity and a healthy diet.

**Methods:**

Participants between 18 and 30 years old residing in Belgium and interested in a healthy diet and/or physical activity, will be recruited. The study will be a mixed-methods research study based on an adapted personal project analysis for goal elicitation, goal appraisal, and rating of inter-goal conflicting or facilitating relations on a cross-impact matrix. The main objectives include examining the conflicting and facilitating relations between health goals and personal values. Secondary objectives include: examining correlations between horizontal and vertical goal relations; and the goal self-concordance score as a method of data triangulation of facilitating relations between goals and personal values.

**Discussion:**

This study will provide insights into how the emerging adult population relate healthy behaviors, specifically physical activity and a healthy diet, to their personal values. The degree to which individuals are able to pursue a health goal is also influenced by other life goals, and therefore the conflicting and facilitating relations between health goals and other life goals will also be examined. This study contributes to multiple health behavior change theories and has implications for the formulation of interventions for the promotion of healthy behaviors.

**Supplementary Information:**

The online version contains supplementary material available at 10.1186/s40359-022-00931-4.

## Background

The incidence of non-communicable diseases (NCDs) such as cardiovascular diseases, diabetes, cancers, and chronic respiratory diseases has increased throughout the twentieth and twenty-first centuries [1]. According to the World Health Organization (WHO), NCDs lead to 71% of global deaths each year, with most deaths occurring in low and middle-income countries [2]. Behavioral risk factors for NCDs, which accounted for 30.3% of disability adjusted life years (DALYS) [3], include physical inactivity, unhealthy diet, tobacco smoking, and alcohol use [2].

Public health interventions have focused on the promotion of healthy lifestyles to prevent and reduce the burden of NCDs [4, 5]. Although most health promotion interventions have targeted a single health behavior [6], a healthy lifestyle with more than one health behavior results in a greater reduction in all-cause mortality [7]. A systematic review of multiple health behavioral change (MHBC) programs showed that interventions varied in their effectiveness [8]. One plausible reason for the mixed findings in MHBC interventions is the daily pursuit of multiple goals. Goals are mental representations of desired outcomes to which people are committed [9]. Setting specific goals increases the chance of reaching the desired outcomes. Self-regulation theories describe the processes people use to help achieve and maintain their goals [9]. The daily pursuit of multiple goals can impede overall progress in the achievement of health goals [10]. For example, a systematic review showed that pursuing the goals of studying or watching TV was negatively associated with physical activity (PA) goals [10]. Goals can also have facilitating relationships, such that accomplishing one goal facilitates reaching another [11]. For example, going to school by cycling fulfills both goals of attending school while performing PA [12].

Another reason for the heterogeneity of findings regarding MHBC interventions is assuming that individuals value health for similar reasons [13]. A review on MBHC interventions indicated that there are differences in how people value health, and therefore health promotion interventions should be individually tailored in terms of the personal values that motivate people in pursuing their health goals [13]. Personal values regarding health differ across a person’s life course. For example, although adolescents recognize that a healthy diet and PA promote health benefits, they value a sense of autonomy in their choices, experiencing new and stimulating challenges, as well as engaging in activities similar to those of their peers.

Since health behavior is influenced by the pursuit of multiple goals, and by the alignment of these goals with personal values, this study will examine the relations amongst personal goals, and between personal goals and values. The present study will use a matrix method, the personal project analysis (PPA) [14] in assessing conflicting and facilitating relations between health goals and other life goals, in addition to understanding which health goals align with an individual’s personal values. The study population will include the age group 18–30 years old, referred to as emerging adulthood, the transition period from adolescence to adulthood that is characterized by an increase in unhealthy behaviors [15].

### Relations between goals and values

**Fig. 1:**
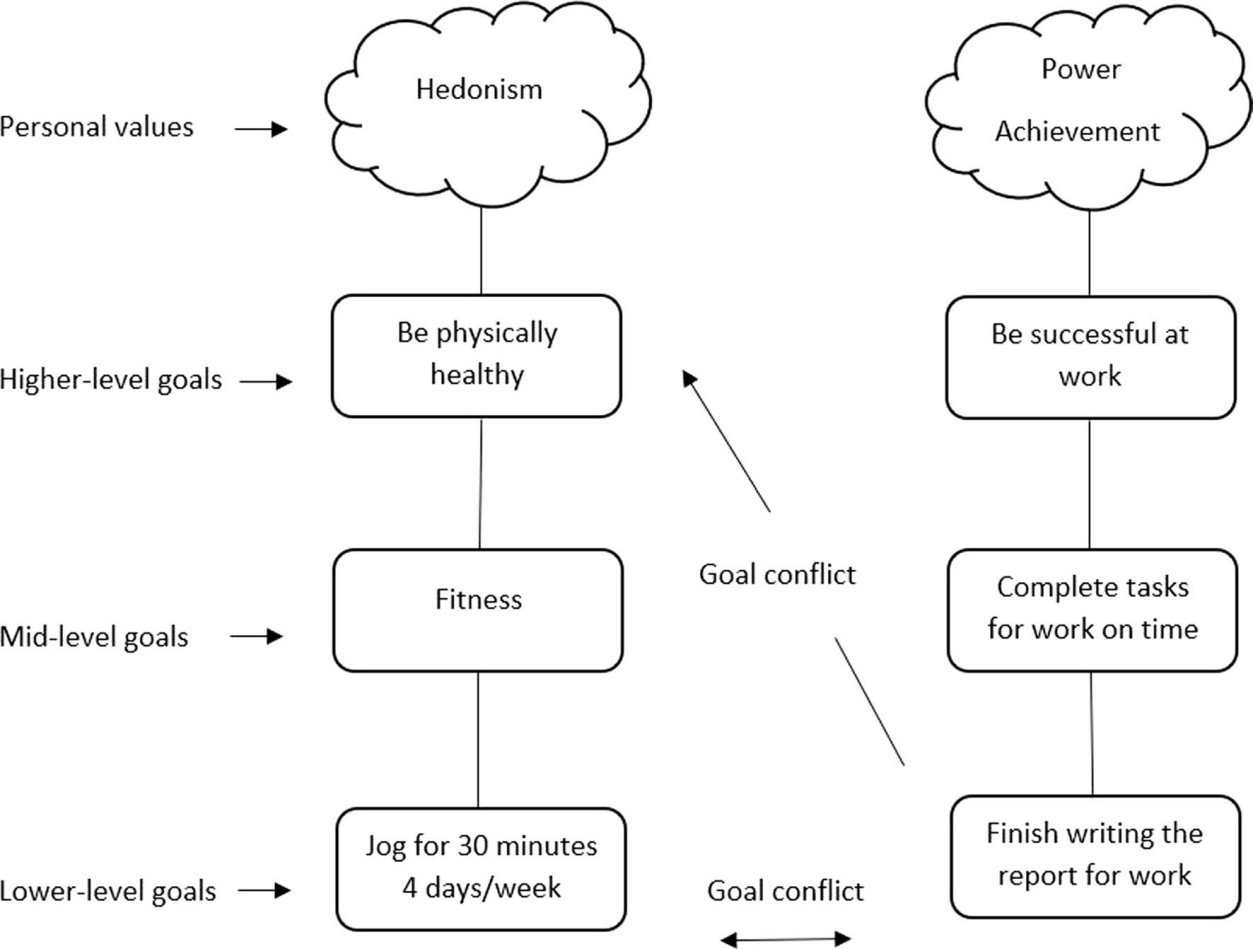
Goal hierarchy diagram

Most research depicts goals as having a hierarchical structure; goals that are behavior-based, such as exercising, are termed lower-level goals and are completed to achieve higher-level goals, such as being physically healthy, which reflect an individual’s personal values (Fig. 1) [16]. The most abstract goals at the highest level of the hierarchy are depicted as personal, general values (e.g., benevolence, achievement, tradition) [16, 17]. Personal values serve as a guide to how individuals can progress towards their goals through goal setting [16]. Mid-level goals are those that are performed midway towards reaching higher-level goals [16]. For instance, one completes a lower-level goal of performing PA, to reach the mid-level goal of fitness and ultimately achieving the higher-level goal of being physically healthy (Fig. 1).

The relations between goals at different levels of a goal hierarchy influence goal engagement. Goals can be conflicting, facilitating, or neutral (i.e., no conflicting or facilitating relations) [16]. Conflicting goals negatively impact well-being [18, 19] and goal progress [10, 19]. Riediger and Freund [11] define goal conflict as when the pursuit of one goal reduces the likelihood of success in reaching another goal. Conflict can occur between lower-level goals, which is referred to as horizontal interference, e.g., not being able to perform regular PA due to work commitments (Fig. 1), and which reduces an individual’s overall progress towards higher-level goals [16]. Conflict can also occur between lower-level goals and higher-level goals, which is referred to as vertical interference, and can be the case when a goal that is pursued is not linked to a personal value [16, 20]. For example, vertical interference occurs when the lower-level goal of finishing a report for work does not help in being physically healthy and conflicts with the value of hedonism (Fig. 1).

Goal facilitation occurs when the achievement of one goal facilitates the achievement of another [11], and has been shown to improve well-being [18, 21] and overall goal progress [10, 21, 22]. Goal facilitation can occur between lower-level goals (i.e., horizontal facilitation) or between lower and higher-level goals (i.e., vertical facilitation) [16]. Horizontal facilitation can be achieved through overlapping goal attainment strategies (progressing in two goals simultaneously: e.g., attending dance class to socialize and learn how to dance) [11], or instrumental goal relations (one goal is instrumental to achieve another: e.g., working to make an income that will facilitate participation in physically active hobbies) [11, 12]. One example of vertical facilitation is completing the lower-level goal of jogging 30 min 4 days/week to reach the higher-level goal of being physically healthy (Fig. 1).

Personal values guide an individual’s attitudes, beliefs, and norms which in turn influence behavior [23]. For example, people who value the environment direct their attention to pro-environmental goals [23]. Examining the relations between goals at different levels of the hierarchy enables us to understand how lower-level health goals are associated with personal values (vertically conflicting or facilitating relations). Emphasizing facilitating relations between lower-level health goals and a personal value may increase an individual’s engagement in healthy behaviors. Individuals who pursue goals that are self-concordant (reflect their interests and personal values) are more likely to succeed in attaining these goals [24]. Additionally, exploring associations between vertical and horizontal goal relations would provide insight into how vertical goal relations influence lower-level goal engagement. For example, individuals who value health goals (high degree of facilitating relations between lower-level health goals and personal values) direct their attention and resources to accomplishing their lower-level health goals (experience low levels of horizontal goal conflict with their health goals) [20].

### Matrix methods in studying inter-goal relations

Interventions exist that focus on personal values in promoting healthy behaviors [25–27]. However, these interventions do not explore the relations between goals within a hierarchy in identifying the factors that influence goal engagement. For example, individuals may value their health (vertical facilitation) but not have time to exercise due to time constraints (horizontal conflict). Studies have explored horizontal relations only between health goals and other life goals using matrix methods [11, 28–30], one of the most used methods in research on inter-goal conflicting and facilitating relations [31]. Two examples of these matrix methods are the Striving Instrumentality Matrix (SIM) [32] and the PPA [14]. In measuring horizontal goal relations, both methods use a semi-structured interview to elicit from participants their personal goals. Participants then rate the degree of conflict or facilitation between pairwise combinations of goals in a matrix [14, 20, 32].

Differences arise between the SIM and PPA in the method of rating pairwise combinations of goals. SIM measures inter-goal relations on a bipolar scale [33] that considers goal conflict and facilitation as opposite ends on a continuum where the impact of one goal on another is rated on a Likert scale from ‘very harmful’ to’very helpful’ [34]. This matrix method does not consider that one goal can have simultaneous conflicting and facilitating relations with another goal [33]. For example, exercising can facilitate the achievement of work goals through relieving stress and therefore improve work efficiency; however, exercising can also interfere with work goals when it takes away time that could be spent working [11]. There have been ambiguities in the interpretation of the SIM rating scale; low ratings may be more representative of facilitative rather than conflicting relations [31]. Therefore, interpretations of the rating scale have led to mixed findings in measuring the influence of inter-goal relations on well-being and goal progress [11].

PPA on the other hand measures inter-goal relations on a unipolar scale that considers goal conflict and facilitation as two different dimensions where one goal can be conflicting another goal in some respects, but also facilitating in others [33]. For example, goal A (work tasks) is rated on how much it conflicts with goal B (exercising) on a Likert scale from 0 (does not conflict at all) to 10 (conflicts a lot), and how much it facilitates goal B from 0 (does not help at all) to 10 (helps a lot) [12, 29]. In rating horizontal inter-goal relations, PPA asks participants to list their personal projects defined as ‘extended sets of personally salient action’ representative of mid-level goals [14]. Participants then rate the conflicting and facilitative relations between these goals (14).

To the best of our knowledge, no study has yet used PPA to measure vertical inter-goal relations. Given that the SIM method has been criticized as a bipolar measure of inter-goal relations, this study aims to use PPA as a unipolar measure of goal conflict and facilitation that can yield more valid results than SIM in measuring vertical inter-goal relations, through exploring how a health goal can be conflicting with a personal value in some situations, or facilitating a personal value in other situations. This method would also complement and provide a more systematic approach in measuring vertical inter-goal relations for other interventions that focus on personal values for health promotion (e.g., motivational interviewing) [25, 26]. Lower-level goals specifically, will be rated in how much they facilitate or conflict with a personal value. In most research that uses PPA to measure inter-goal relations, the abstraction level of the goals in the hierarchy is not specified, and therefore ambiguities remain at which level of the goal hierarchy conflict or facilitation is being measured [12, 18, 28, 29, 35]. As a first step in PPA the participants are asked to list their personal projects. The adaptation of this PPA protocol lies in eliciting lower and higher-level goals from the participants’ projects prior to completing the cross-impact matrix. Lower-level rather than mid-level goals were chosen for assessing inter-goal relations because conflict is more likely to occur between goals that are action-based (lower-level goals) [16].

Participants then match their higher-level goals to relevant personal values from a list. This approach would familiarize participants with the list of personal values, while at the same time allowing them to be more introspective about their reasons for engaging in their lower-level goals. Afterwards, participants complete the cross-impact matrix to rate the horizontal goal relations (conflict and facilitation between lower-level goals), and the vertical goal relations (conflict and facilitation between lower-level goals and the full list of personal values). This method would allow for an examination of relations between goals at different levels of the hierarchy, and identification of discrepancies between lower-level goals and personal values, while reflecting the conflicting or facilitating goal relations that participants experience.

### Study aims

An adapted version of the PPA will be developed to evaluate the following:

- Conflicting and facilitating relations between lower-level goals (horizontal goal relations) and between lower-level goals and personal values (vertical goal relations). We will focus on the lower-level health goals of PA and a healthy diet.
- Lower-levels goals are not restricted to healthy diet and PA, and are elicited in an open manner using a semi-structured interview. However, the focus of our study is on healthy diet and PA, and participants will be recruited such that they are likely to have at least one PA or healthy diet lower-level goal.
- Rating of lower-level goals against goal dimensions (goal importance, difficulty, competence, stress). The PPA has been used to appraise goals on certain dimensions such as goal difficulty and goal importance [14]. Goal appraisals have been examined in relation to goal content (e.g., goals focused on education are appraised as low in control), and well-being (e.g., persons with high levels of depression appraised their goals as low in achievement) [36]. In this study, associations between vertical goal conflict or facilitation and goal appraisals will be explored (e.g., do individuals who score high in vertical goal conflict appraise their goals as more difficult?).
- A measure of the participants’ self-concordance will be included as a method of data triangulation to validate the findings of the vertical facilitation measure. Self-concordance represents the degree to which a goal matches a person’s values [20, 37, 38]. Individuals with higher self-concordance pursue goals that reflect their values and interests [20, 37, 38].

## Objectives

### Main objectives

- Explore associations between lower-level health goals and personal values; what are the conflicting and facilitating relations between lower-level goals (with a specific focus on a healthy diet and PA) and personal values? For example, does PA facilitate the personal value of achievement more than a healthy diet?

### Secondary exploratory objectives

- How do different lower-level goals (specific to PA and diet) score on goal dimensions (e.g., goal importance, goal difficulty)? For example, we want to determine if PA is considered more difficult or more important than diet.
- What is the association between the average rate of goal conflict and facilitation of PA/diet with other goals, and PA/diet goal dimensions? For example: If PA is highly conflicting with other lower-level goals, does PA also score higher on difficulty?
- Is goal self-concordance positively associated with a global average score of vertical goal facilitation?
- What are the associations between horizontal and vertical inter-goal conflict and facilitation?
- Qualitative thematic analysis of the types of goals that individuals pursue. These data can be used for item construction for future studies.

## Methods

### Research design

This study will be a cross-sectional mixed-method study with a combination of qualitative and quantitative research methods, based on the PPA method by Little [14, 39]. In line with previous studies that used the PPA, we will conduct a semi-structured interview to elicit projects and explore the participants’ higher and lower-level goals [28, 40, 41]. The participants will then proceed with goal appraisal (rating their lower-level goals against goal dimensions) and use the matrix to rate conflicting and facilitating relationships amongst their lower-level goals, and between their lower-level goals and personal values (see Additional file 1: Appendix A).

### Sampling method and recruitment

We will recruit participants via a convenience sample of employment and leisure organizations, taking socio-economic (e.g., white/blue collar jobs), regional (rural/urban) and potential gender-based differences into account. We will also recruit through personal networks, and an online platform where students participate in research to gain course credits.

### Inclusion and exclusion criteria

The inclusion criteria are a general healthy adult male and female population aged 18–30 years old, who understand Dutch, French, or English, and are currently residing in Belgium. We focus on this age group because emerging adults undergo many life changes that influence their health behaviors [15, 42]. Because we are focusing in our study on lower-level health goals, we will recruit participants who intend to engage in PA and/or a healthy diet, or are already doing so.

The exclusion criteria are individuals who are physically unable to perform certain healthy lifestyles such as those unable to walk for at least 100 m, following a medically restricted diet, or those currently undergoing any treatment that impacts healthy lifestyles (for example chemotherapy, in relation to diet). We will also exclude those with current or a history of eating disorder, and not able to read Dutch, French or English.

### Sample size calculation

We will need a sample size of 82 (G*Power: medium effect size, α = 0.05, 1 − β = 0.80) to find moderate effect sizes. This sample size is based on the correlation analyses between horizontal and vertical goal relations. Sample sizes for other analyses include: t-tests for differences between PA and diet in goal appraisals (difference between two dependent means, matched pair; required sample size n = 34), and ANOVA for differences between PA and diet in rating conflicting and facilitating relations with personal values (repeated measures, within-between interactions; required sample size *n* = 18).

### Procedure

**Fig. 2:**
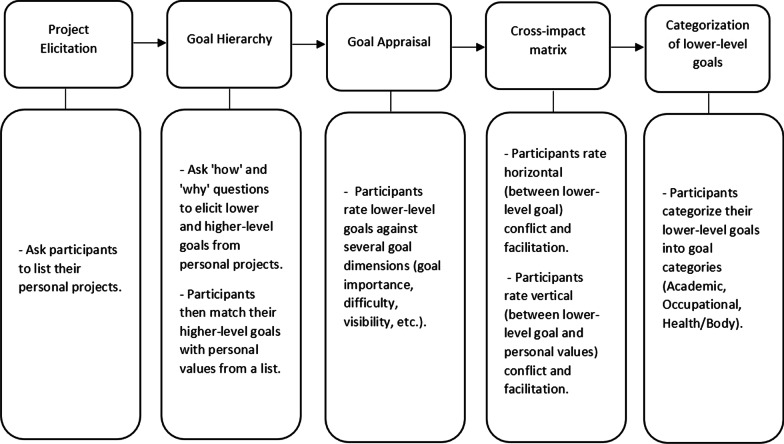
Diagram for conducting steps of the adapted Personal Project Analysis

The PPA will initially be completed with the participants as a semi-structured interview to be scripted and audio-recorded. The PPA procedures will be as follows: project elicitation; goal hierarchy; goal appraisal; cross-impact matrix; and categorization of lower-level goals (Fig. 2). In project elicitation, researchers elicit from the participants their personal projects. In completing the goal hierarchy, the participants are then asked to generate their lower and higher-level goals from their personal projects, and the higher-level goals are categorized by the participants into personal value categories from a list. Afterwards, the participants rate their lower-level goals according to a list of goal dimensions. The participants then complete cross-impact matrices to rate horizontal and vertical inter-goal conflicting and facilitating relations. The last step of the PPA is categorizing their lower-level goals into goal categories. After completing the PPA, participants will be administered the self-concordance scale and a baseline survey to collect information on their socio-demographic variables (age, gender, socio-economic status). The PPA components will be written in lay terms for the participants to have an easier understanding of the questionnaire. Lower-level goals will be referred to as ‘Actions’, and higher-level goals as ‘Personal values.’

### The personal project analysis

#### Project elicitation

Researchers will elicit the participants’ personal projects. We will ask them to list between 3–8 personal projects. Previous studies that had elicited personal projects with no restriction on the number indicated that participants listed an average of 5.76 goals (SD = 2) with a range of 3–12 goals [40], and an average of 6.19 goals in another study [12]. Based on these studies and previous ones that elicited a maximum of 8 projects [43–45], we will limit the minimum number of projects to 3, and a maximum of 8. Participants are given a description and examples of personal projects based on Little’s approach [14]. Current or upcoming planned projects are emphasized to make the projects representative of the participants’ actual pursuits [14].

#### Goal hierarchy: exploring higher and lower-level goals

In the goal hierarchy step, researchers explore the participants’ lower and higher-level goals. Little assumes that personal projects are mid-level goals [14]. Based on Little’s method, the researchers can ask the participants ‘how’ and ‘why’ questions to elicit higher and lower-level goals from the personal projects [14]. To ensure the same level of concreteness for the lower-level goals, we will ask ‘how’ questions to reach action-based goals that represent completing an activity (e.g., help my friend move out), and remain relevant over time. For the higher-level goals, we will ask ‘why’ questions until we reach ‘be goals’ (e.g., be a good friend). The participants will then match their higher-order goals with a list of personal values by Schwartz [17]. We selected this list of values because it is one of the most representative of universal values [17].

#### Goal appraisal

We will ask participants to rate their lower-level goals against a list of goal dimensions that will include goal importance, difficulty, competence, and stress. We chose these goal dimensions because they reflect different aspects of goal pursuit such as meaning (e.g., importance), stress (difficulty, stress), and efficacy (e.g., competence) [14, 46].

#### Cross-impact matrix

The participants will complete four matrices to rate horizontal and vertical inter-goal relations. Inter-goal conflict and facilitation will be measured independently in each matrix. The first two matrices will measure horizontal goal conflict and facilitation between lower-level goals, and the last two will measure vertical goal conflict and facilitation between lower-level goals and personal values. For horizontal goal matrix ratings, the lower-level goals will be listed on the either side of the matrix, so that each pairing of goals will be rated against the other. Whereas in rating the vertical inter-goal relations, the lower-level goals will be listed across the vertical side of the matrix, and the personal values will be listed on the horizontal side of the matrix. Accordingly, lower-level goals will be rated in their conflicting or facilitating relations with the full list of personal values (10 values). The Likert scale for assessing goal conflict will go from 0 (not difficult at all) to 5 (very difficult), and the scale for goal facilitation will go from 0 (does not help at all) to 5 (very much help) [29].

#### Goal categorization

After completing the cross-impact matrices, participants will categorize their lower-level goals according to a goal categorization (e.g., academic, occupational, health/body) (40).

### Self-concordance ratings

After completing the PPA, the participants will complete the goal self-concordance measure. For each participant, a self-concordance score will be calculated by summing the intrinsic and identified ratings and subtracting them from the external and introjected ratings [47].

### Data analysis

Data will be analyzed using R version 4.1.2 [48]. Descriptive analysis of the data will be completed. We will report the average number of goals reported by participants and calculate the percentages of goal categories to obtain a frequency of the goal types. We will also count the number of times a PA or diet goal was chosen for a personal value to obtain frequencies for the type of health goal chosen for a personal value. We will also complete a qualitative thematic analysis describing the participants’ objectives.

#### Goal appraisal

Descriptive statistics (mean, SD) for goal appraisal will be calculated. Pairwise t-tests will be completed to assess differences in how individuals appraise their lower-level goals (PA vs healthy diet) with respect to their goal dimensions. Correction for multiple comparisons will be applied when needed.

#### Inter-goal relations

Our focus in the cross-impact matrix is on the lower-level goals of PA and a healthy diet. Percentages of the sample that report their PA or healthy diet goals as conflicting or facilitating will be calculated. Descriptive statistics (mean, SD) will be calculated for the matrices of vertical and horizontal inter-goal relations. ANOVAs will be completed to explore for differences between personal values in how much they conflict or facilitate the lower-level goals of PA and a healthy diet.

We will also investigate for correlations between the following: horizontal and vertical inter-goal relations; goal dimensions and vertical inter-goal relations; and vertical facilitation and self-concordance.

## Discussion

The personal goals that individuals set are influenced by their personal values. The extent to which an individual values achievement for example, determines the efforts that will be directed towards occupational goals. At the same time, an individual’s ability to accomplish a goal is influenced by personal resources (energy, financial resources, time, pursuit of other life goals). This study will explore how individuals value healthy behaviors, specifically PA and a healthy diet. The study will also draw associations between personal values and health goals, and how it relates to other life goals (conflicting or facilitating other life goals and vice versa). The study will contribute to understanding the factors that influence engagement in multiple healthy behaviors, especially in the emerging adult population. The study will also contribute to existing interventions that are focused on personal values in promoting healthy behaviors, such as motivational interviewing.

## Supplementary Information

**Additional file 1.** Description of the Personal Project Analysis Protocol used to collect data.

## Data Availability

The datasets used and/or analysed during the current study are available from the corresponding author on reasonable request.

## Abbreviations

NCDs: Non-communicable diseases
WHO: World health organization
DALYS: Disability adjusted life years
MHBC: Multiple health behavioral change
PA: Physical activity
PPA: Personal project analysis
SIM: Striving instrumentality matrix
ANOVA: Analysis of variance
