# Supplementary material for: Study protocol for an adapted personal project analysis to measure vertical inter-goal relations on physical activity and diet
Source: BMC Psychol. 2022 Sep 24;10:225. doi: 10.1186/s40359-022-00931-4 (PMC9509544; doi:10.1186/s40359-022-00931-4)
Supplement: Supplementary file 1 — Additional file 1. Description of the Personal Project Analysis Protocol used to collect data. [file 40359_2022_931_MOESM1_ESM.docx]

**Appendix A: Personal Project Analysis study:**

1. **Project elicitation:**

We are interested in studying the kinds of activities and concerns that people have over the course of their lives. We call these personal projects. All of us have a number of personal projects at any given time that we think about, plan for, carry out, and sometimes (though not always) complete.

Some projects may be focused on achievement (getting my degree) others on the process (enjoying a night out with the friends); they may be things we choose to do or things we have to do; they may be things we are working towards or things we are trying to avoid. Projects may be related to any aspect of your daily life, university, work, home, your-self, relationships, leisure, and community, among others. Please think of projects in this broad way.

Some examples of personal projects:

- Pass my courses.

- Cut down on junk food.

- Play with my cat.

- Clean my house.

- Try to get along well with my family members.

- Act according to my religious or ideological beliefs.

- Exercise more often.

- Plan my summer vacation.

- Be a better parent.

- End a relationship with a partner.

- Run a marathon.

- Show more understanding towards a friend.

- Find a new job.

- Stop put studying off until the last minute.

To start, we will take 10-15 minutes to fill in the following cells 3-8 personal projects and activities that you are currently engaged in or considering – remember these need not be formal projects or even important ones – we would prefer you to give us more of the everyday kinds of activities or concerns that characterize your life at present. These projects should also be relevant over time. Please be completely honest in your answers, as they will be not connected to your name.

| **Project #** | **Project Description** |
| --- | --- |
| 1. | Complete my tasks for work |
| 2. | Cook more meals at home |
| 3. | Plan more trips with my partner |
| 4. |  |
| 5. |  |
| 6. |  |
| 7. |  |
| 8. |  |

1. **Exploring how you complete your projects and value them:**

We want to look at how you try to do your projects, and why these projects are important for you to achieve (e.g., security, achievement). Afterwards we will match your reasons for these projects with a list of personal values. Place your mouse cursor on the personal value definitions, and the personal value meanings will pop up. You can match your reasons for these projects with more than one personal value. If you do not find a personal value in the list, then you can add your own personal value.

| **Project #** | **Personal Project** | **Action (how ?)** | **Personal value (why ?)** | **Personal value categorization** | **Personal value definitions** |
| --- | --- | --- | --- | --- | --- |
| 1. | Complete my tasks for work | Complete writing the report on time | Do well at work | Achievement |  |
| 2. | Cook more meals at home | -- | Maintain a healthy diet | Be more healthy |  |
| 3. | Plan more trips with my partner | -- | Enjoy my leisure time more | Hedonism - Benevolence |  |
| 4. |  |  |  |  |  |
| 5. |  |  |  |  |  |
| 6. |  |  |  |  |  |
| 7. |  |  |  |  |  |
| 8. |  |  |  |  |  |

Personal value definitions:

POWER: Social recognition, Preserving my public image, Authority, Wealth, Social power, Acknowledgement, Power, Prestige, Status, Affluence.

ACHIEVEMENT: Intelligence, Capability, Success, Ambition, Being influential, Influence, Competition, Assertivity, Goal orientation, Purposefulness.

HEDONISM: Pleasure, Enjoying life, Satisfaction, Being happy, Bliss, Amusing oneself, Fun.

STIMULATION: Daring, Stimulating life, Variety, Change, Adventure, Experimenting.

SELF-DIRECTION: Independence, Freedom, Curiosity, Creativity, Choosing own goals, Self-respect, Autonomy, Originality, Being unique, Individualism.

UNIVERSALISM: Broadmindedness, Equality, Unity with nature, Inner harmony, Protecting nature, World of beauty, Wisdom, World peace, Justice, Responsibility, Making the world a better place, Societal improvement, Fairness, Insight.

BENEVOLENCE: Forgiving, Honesty, Helpfulness, Loyalty, Love, Friendship, Support, Assistance, Helping, Carefulness, Care, Compassion, Friendliness, Truth, Truthfulness.

TRADITION: Modesty, Piety and devotion, Tradition, Moderation, Steadiness, Virtue.

CONFORMITY: Obedience, Respect for parents/elders, Politeness, Self-discipline, Sense of duty, Dutifulness.

SECURITY: Protection/security of family, Social order, Cleanliness, Reciprocity, Belongingness, National security, Stability, Certainty, Social security.

1. **Appraisal of actions relevant to projects:**

Please rate each action below from 0-5 on the series of dimensions listed above them. Place your mouse cursor on the dimension name and the dimension meaning will pop up.

| **Action #** | **Actions:** | **Importance** | **Difficulty** | **Competence** | **Stress** |
| --- | --- | --- | --- | --- | --- |
| 1. | Complete writing the report on time | 5 | 5 | 4 | 5 |
| 2. | Cook more meals at home | 5 | 4 | 3 | 5 |
| 3. | Plan more trips with my partner | 4 | 3 | 3 | 4 |
| 4. |  |  |  |  |  |
| 5. |  |  |  |  |  |
| 6. |  |  |  |  |  |
| 7. |  |  |  |  |  |
| 8. |  |  |  |  |  |

**Definitions of dimensions:**

- Importance: How important is this action for you?

Use 5 if it is very important and 0 if it is not at all important.

- Difficulty: How difficult do you find it to carry out this action?

Use 5 for an action that is extremely difficult to carry out, and 0 for an action that is not difficult at all.

- Competence: To what extent do you feel competent to carry out this action?

Use 5 if you feel completely competent to carry out this action, and 0 if you do not feel competent to carry it out.

- Stress: How stressed do you feel while engaged or thinking about each action?

Use 5 if you feel completely stressed about this action, and 0 if you do not feel stressed at all.

1. **Cross-impact matrix**

Please rate from 0-5 the extent to which actively engaging in each of these actions makes it difficult to engage in the other actions

0: not difficult at all

5: very difficult

0------------------------1------------------------2------------------------3--------------------------4--------------------------5

Not difficult at all Very little difficulty Little difficulty Somewhat difficult Moderately difficult Very difficult

|  | | **Actions:** | | | | | | | | | |
| --- | --- | --- | --- | --- | --- | --- | --- | --- | --- | --- | --- |
| **Action #** | **Actions:** | Complete writing the report on time | Cook more meals at home | Plan more trips with my partner |  |  |  |  |  |  |  |
| 1. | Complete writing the report on time |  | 5 | 5 |  |  |  |  |  |  |  |
| 2. | Cook more meals at home | 5 |  | 2 |  |  |  |  |  |  |  |
| 3. | Plan more trips with my partner | 5 | 3 |  |  |  |  |  |  |  |  |
| 4. |  |  |  |  |  |  |  |  |  |  |  |
| 5. |  |  |  |  |  |  |  |  |  |  |  |
| 6. |  |  |  |  |  |  |  |  |  |  |  |
| 7. |  |  |  |  |  |  |  |  |  |  |  |
| 8. |  |  |  |  |  |  |  |  |  |  |  |

Please rate from 0-5 the extent to which actively engaging in each of these actions helps with engaging in the other actions.

0: does not help at all

5: very much help

0------------------------1------------------------2------------------------3--------------------------4--------------------------5

Does not help at all Very little help Little help Somewhat helps Moderately helps Very much help

|  | | **Actions:** | | | | | | | | | |
| --- | --- | --- | --- | --- | --- | --- | --- | --- | --- | --- | --- |
| **Action #** | **Actions:** | Complete writing the report on time | Cook more meals at home | Plan more trips with my partner |  |  |  |  |  |  |  |
| 1. | Complete writing the report on time |  | 0 | 0 |  |  |  |  |  |  |  |
| 2. | Cook more meals at home | 0 |  | 0 |  |  |  |  |  |  |  |
| 3. | Plan more trips with my partner | 4 | 0 |  |  |  |  |  |  |  |  |
| 4. |  |  |  |  |  |  |  |  |  |  |  |
| 5. |  |  |  |  |  |  |  |  |  |  |  |
| 6. |  |  |  |  |  |  |  |  |  |  |  |
| 7. |  |  |  |  |  |  |  |  |  |  |  |
| 8. |  |  |  |  |  |  |  |  |  |  |  |

Please rate from 0-5 the extent to which actively engaging in each of these actions makes it difficult to realize your personal values

0: not difficult at all

5: very difficult

0------------------------1------------------------2------------------------3--------------------------4--------------------------5

Not difficult at all Very little difficulty Little difficulty Somewhat difficult Moderately difficult Very difficult

|  | | **Personal values:** | | | | | | | | | |
| --- | --- | --- | --- | --- | --- | --- | --- | --- | --- | --- | --- |
| **Action #** | **Actions:** | Power | Achievement | Hedonism | Stimulation | Self-direction | Universalism | Benevolence | Tradition | Conformity | Security |
| 1. | Complete writing the report in time | 0 | 0 | 3 | 1 | 0 | 0 | 2 | 0 | 0 | 0 |
| 2. | Cook more meals at home | 0 | 3 | 0 | 0 | 0 | 0 | 0 | 0 | 0 | 0 |
| 3. | Plan more trips with my partner | 0 | 0 | 0 | 0 | 0 | 0 | 0 | 0 | 0 | 0 |
| 4. |  |  |  |  |  |  |  |  |  |  |  |
| 5. |  |  |  |  |  |  |  |  |  |  |  |
| 6. |  |  |  |  |  |  |  |  |  |  |  |
| 7. |  |  |  |  |  |  |  |  |  |  |  |
| 8. |  |  |  |  |  |  |  |  |  |  |  |

Please rate from 0-5 the extent to which actively engaging in each of these actions helps you to realize your personal values

0: does not help at all

5: very much help

0------------------------1------------------------2------------------------3--------------------------4--------------------------5

Does not help at all Very little help Little help Somewhat helps Moderately helps Very much help

|  | | **Personal values:** | | | | | | | | | |
| --- | --- | --- | --- | --- | --- | --- | --- | --- | --- | --- | --- |
| **Action #** | **Actions:** | Power | Achievement | Hedonism | Stimulation | Self-direction | Universalism | Benevolence | Tradition | Conformity | Security |
| 1. | Complete writing the report in time | 2 | 5 | 2 | 4 | 5 | 4 | 0 | 0 | 3 | 5 |
| 2. | Cook more meals at home | 0 | 2 | 3 | 3 | 0 | 0 | 5 | 0 | 0 | 5 |
| 3. | Plan more trips with my partner | 0 | 4 | 5 | 5 | 0 | 0 | 5 | 0 | 0 | 3 |
| 4. |  |  |  |  |  |  |  |  |  |  |  |
| 5. |  |  |  |  |  |  |  |  |  |  |  |
| 6. |  |  |  |  |  |  |  |  |  |  |  |
| 7. |  |  |  |  |  |  |  |  |  |  |  |
| 8. |  |  |  |  |  |  |  |  |  |  |  |

1. **Categorization of action:**

Please categorize your actions according to the drop down list in the 'Category' section. Definitions of the categories can be found when you move your cursor over 'Category definitions’. You can add more than one category per action.

| **Action #** | **Your actions:** | **Category:** | **Category definitions:** |
| --- | --- | --- | --- |
| 1. | Complete writing the report in time | Occupational |  |
| 2. | Cook more meals at home | Health/body |  |
| 3. | Plan more trips with my partner | Leisure - Interpersonal |  |
| 4. |  |  |  |
| 5. |  |  |  |
| 6. |  |  |  |
| 7. |  |  |  |
| 8. |  |  |  |

**Category definitions:**

- Academic:

School/university-related.

For example: get my teaching certificate - study harder for exams.

- Occupational:

Job-related such as job tasks or job-related courses.

For example: Find a more rewarding career - finish inventory by Tuesday.

- Physical activity:

Physical activity includes all large body movements (e.g., moving your legs and/or arms), such as walking, running, cycling, doing gardening, household chores such as mopping floors or cleaning windows, heavy lifting, swimming, sports, …. Some physical activities will make your heart beat a bit faster and may make you out of breath, but they can also include calmer, lighter activities

- Diet:

Actions that you take to have a healthy diet, which may include eating a varied diet, eating sufficient fruit and vegetables, eating sufficient grains and nuts, limiting meat and other fat consumption, limiting sugar consumption, drinking water instead of sugar-sweetened beverages, not eating too much salt.

- Other health and body:

Activities related to health that do not include physical activity and diet.

For example: monitoring medication – improve my sleep.

- Interpersonal:

Actions dealing with others on a personal level, includes family, friends, and intimate others.

- Intrapersonal:

Actions dealing with outlook and attitudes relating to the self, including self-improvement, spiritual, philosophical, or activities related to coping, or adjustment.

For example: stop being so anti-social - work on my self-esteem.

- Leisure:

Recreational activities done alone or with others. For example: Go bungee jumping with Mike - Read more for pleasure

- Maintenance:

Activities relating to organization and administration, including household and financial maintenance activities, pet maintenance, paperwork, etc.

For example: Clean out the basement - Get the car tuned up.
